# Afforestation of *Taxodium* Hybrid Zhongshanshan Influences Soil Bacterial Community Structure by Altering Soil Properties in the Yangtze River Basin, China

**DOI:** 10.3390/plants11243456

**Published:** 2022-12-09

**Authors:** Qin Shi, Zhidong Zhou, Ziyang Wang, Zhiguo Lu, Jiangang Han, Jianhui Xue, David Creech, Yunlong Yin, Jianfeng Hua

**Affiliations:** 1 Jiangsu Key Laboratory for the Research and Utilization of Plant Resources, Institute of Botany, Jiangsu Province and Chinese Academy of Sciences (Nanjing Botanical Garden Mem. Sun Yat-Sen), Nanjing 210014, China; 2 College of Biology and Environment, Nanjing Forestry University, Nanjing 210037, China; 3 Arthur Temple College of Forestry and Agriculture, Stephen F. Austin State University, Nacogdoches, TX 75962-3000, USA

**Keywords:** Yangtze River Basin, *Taxodium* hybrid Zhongshanshan, soil properties, bacterial community structure

## Abstract

*Taxodium* hybrid Zhongshanshan has been widely planted in the Yangtze River Basin (YRB) for soil and carbon conservation, with quantities over 50 million. The objective of this study was to determine how *T*. hybrid Zhongshanshan plantations affected soil physicochemical properties and bacterial community structure in the YRB, and to examine the consistency of changes by afforestation. Soils under *T*. Zhongshanshan plantations across six sites of the YRB were compared with soils of adjacent non-forested sites. Soil physicochemical properties and bacterial community structure were determined to clarify edaphic driving factors and reveal the effects of afforestation on bacteria. The results indicated that most soil attributes manifested improvements, e.g., total nitrogen in Jiangxi and Shanghai; available phosphorus in Hubei, Chongqing and Yunnan, exhibited the potential to maintain or ameliorate soil quality. A decrease in soil bulk density caused by plantation was also observed at the expense of soil macro-aggregates augment. Afforestation of *T*. Zhongshanshan plantation has habitually improved Shannon diversity and Chao1 richness, of which dominant phyla were Proteobacteria, Acidobacteria, and Actinobacteria, and increased the relative abundance of the phyla Proteobacteria and Nitrospirae, and the classes *Flavobacteriia*, *Acidobacteria*_Gp5, and *Bacilli*. We concluded that *T*. Zhongshanshan plantation can be employed to facilitate soil nutrient accumulation in the YRB, but that the degree, rate and direction of changes in soil attributes are sites dependent. It is recommended that afforestation of nutrient-depleted and less productive lands in the YRB should utilize this fast-growing species in combination with proper fertilization.

## 1. Introduction

The Yangtze River Basin (YRB) originates from the east of Qinghai-Tibet Plateau, traverses three major economic zones of eastern, central, and western of China, accounting for 18.8% of China’s land area [1]. Due to the dual effects of climate change and anthropogenic activities, the vegetation in the YRB has degenerated to some extent. In terms of soil and water conservation, a series of large-scale and long-standing afforestation projects have been implemented in this area, such as the Yangtze River Basin Shelterbelt Construction Project, which has greatly increased forest area by 6.02 × 10^5^ km^2^ from 1998 to 2014 [2]. Individual trees are known to alter soil properties, creating a section of influence under the tree canopy [3]. Issues related to the alteration of soil condition caused by tree plantations have aroused concerns for many years [4,5,6]. It was reported that afforestation could affect most soil properties and biological activities: water-retention characteristics, bulk density (BD), particle-size distribution, content of nutrients and microbial properties [7,8]. Practice of afforestation in Xiong’an New Area suggested that mixing of different species helped trees to make the utmost of nutrients and increased soil bacterial diversity [9]. Studying with five common native tree species, Diao et al. [10] proclaimed that native broad-leaved tree species played pivotal roles in maintaining soil chemical and microbial properties in a temperate secondary forest. These changes are owing to understory vegetation existing and the effects of trees themselves.

*Taxodium* hybrid Zhongshanshan is a superior interspecies hybrid of *T*. *distichum* and *T*. *mucronatum*. Due to its impressive water tolerance, fast growth, and adaptability to degraded sites, it has been widely planted in southeastern China and is considered potentially as a universally accepted tree species for building forest cities and realizing carbon neutrality. As one of the main afforestation tree species, *T*. Zhongshanshan was introduced to Chongqing in 2002 and immediately became the eligible species on account of its innate superiorities. The experiment in the Three Gorges Reservoir exhibited a more than 90% survival rate of the *T*. hybrids after they were wholly submerged for an utmost of 122 d under water depth to 12 m [11]. Hitherto, large YRB areas of *T*. Zhongshanshan plantation have been artificially established as ecological forests, with quantities over 50 million. The current *T*. Zhongshanshan stands in the YRB area can store up to 217 t ha^−1^ carbon, and have a substantial potential for carbon sequestration as the total land area under these plantations is consecutively expanding, most of which are still immature [12]. The demand for landscape plantations and timber for this species still outstrips supply.

In recent years, intensive studies concerning the water resistance mechanism of *T*. Zhongshanshan have been carried out [13,14,15], while few studies have quantified the influence of *T*. Zhongshanshan on soil characteristics. To date, no field plantations studies have included microbiology aspects of *T*. Zhongshanshan. A better understanding of the soils of *T*. Zhongshanshan plantations in the YRB differ, and the underlying mechanisms will improve our competence to predict the effects of *T*. Zhongshanshan plantation on the YRB ecosystems, and then guide the further popularization with evidence. Moreover, it is the required first step toward understanding the consequences faced by terrestrial ecosystems and soil biogeochemical cycling. The aims of this paper, which are part of a larger study on afforestation ecology of *T*. Zhongshanshan in the YRB, are as follows: (i) to compare soil physicochemical properties and bacterial community structure under afforested and non-afforested sites; (ii) to examine the consistency of changes in soil properties by afforestation, if any, across sites of the YRB.

## 2. Materials and Methods

### 2.1. Study Location and Site Description

**Figure 1:**
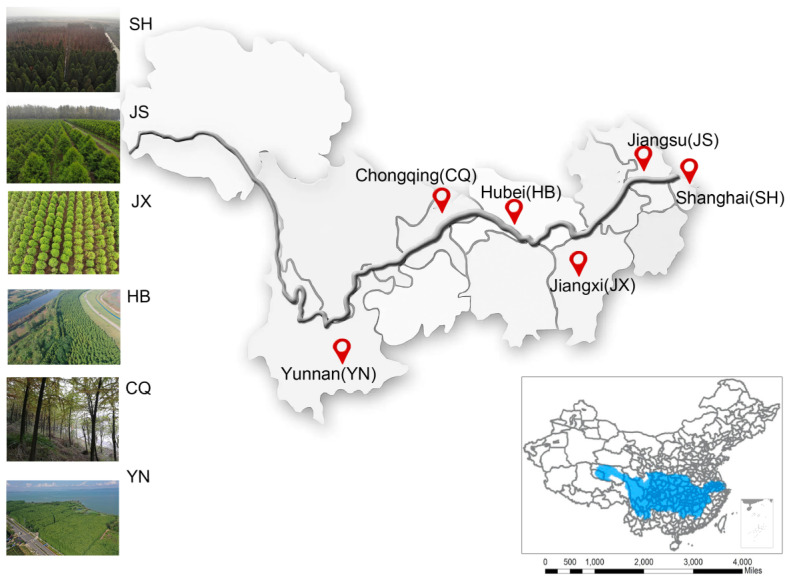
Spatial distribution of 6 *T*. Zhongshanshan stands in the Yangtze River Basin (blue area in the lower right corner).

As the largest basin in China, the YRB locates between 90°33′−122°25′ E and 24°30′−35°45′ N, characterized by a subtropical monsoon climate. We selected afforested plantation areas in 5 Provinces and 1 Municipality with aspects from west to east (102°42′−121°32′ E), and from 1891 to 3 m above sea level, representing the general status of the YRB. The annual average temperature ranges from 12.6 to 18.0 °C and annual average precipitation is about 1086 mm. Details of afforestation sites were given in Figure 1. 

### 2.2. Experiment Design and Soil Sampling

*T*. Zhongshanshan plantations aged sequence of 6, 8, 9, 11, 13, and 14 years in YRB were selected as experimental stands, and all were >10 ha. The vegetation in the plantation areas and adjacent non-afforested sites was composed of *Erigeron annuus* (L.) Pers., *Chenopodium album* L., *Rubus hirsutus* Thunb., *Angelica sieboldin* (F.) hirsutum, *Mazus japonicus* (Thunb.) Kuntze, *Plantago depressa* Willd., *Setaria viridis* (L.) Beauv. and *Solidago canadensis* L., with sparsely scattered woods (mainly *Morus alba* L. and *Ulmus pumila* L.). Basic information for the different stands including coordinate, soil color, tree height, diameter at breast height (DBH), crown diameter (CD) and tree density were recorded in Table 1. Aboveground biomass (AGB) was calculated according to the allometric growth equation elaborated by Shi et al. [12]. Both plantations and the adjacent non-afforested soils had not received any fertilization or weed control treatment. Therefore, the adjacent non-afforested soils could be considered as a control (CK) for this study. Any soil differences here could reflect the effects of the plantation establishment process. The experiment began in June 2021, and lasted for two months. We established large sites with a size of 1 ha covering minor local heterogeneity in soil conditions, which allowed us to differentiate 3 fixed plots of 20 × 30 m in each stand and were spaced 50 m from each other. Therefore, a total of 36 plots were established for soil sample collection.

Litter horizons were removed before soil sampling. Three soil profiles were set and diagonally distributed in each of the 36 plots. A total of 15 soil profiles were manually dug by a shovel from each plot at different arbitrary distances within 0.3–1 m from the tree trunks, in which the vertically projected crown of each *T*. Zhongshanshan was covered. To ensure the sampling points keeping identical from the trunks, we chose 0.5 m away from the trunk as the sampling point. At each plot, soil samples at the depths of 0–15, 15–30, and 30–45 cm were collected from the *T*. Zhongshanshan plantations. Soils within the same depth of three profiles were mixed together as one sample which containing approximately 500 g of soils, and thus a total of 108 samples (3 plots × 3 soil depths respectively in 6 afforested and non-afforested plots) were obtained. Another set of soil samples from 0–15, 15–30, and 30–45 cm depths were separately and intactly collected by inserting a steel cylinder of known volume (5 cm high and with a 5 cm inner diameter) for BD determination. All samples were transported to the laboratory and divided into three parts. Before being air-dried, approximately 10 g of fresh soil samples were disengaged and stored in 4 °C for microbial assays. Part of the remaining air-dried samples in the same profile was mixed thoroughly for soil aggregate distribution determination, and another part was sieved passed through a 2-mm screen for further analyses. 

### 2.3. Laboratory Analysis

Soil pH and electrical conductivity (EC) were measured in a soil-water suspension (1:5 soil-water ratio) by a pH (Yueping PHS-3CU, Shanghai, China) and conductivity meter (Yueping DDS-307, Shanghai, China). After being sieved < 0.25 mm, a total of 1.00 g soils were digested in H_2_SO_4_ with mixed catalyst (K_2_SO_4_:CuSO_4_:Se = 5:1:1, w:w:w) and heated at 400 °C until the contents became colorless. The extracting solution was analyzed using the Kjeldahl method to determine total nitrogen (TN) according to Wu et al. [16]. Available phosphorus (AP) was determined by the molybdenum antimony resistance colorimetry. Samples of 5.0 g soil were extracted in 100 mL of non-phosphorus sodium bicarbonate, and then soil AP was determined using Mo-Sb colorimetric analysis. For soil available potassium (AK) determination, 5.0 g soils were extracted in ammonium acetate and then measured with flame atomic absorption spectrometry (Agilent 240 AA, Santa Clara, CA, USA). After they were sieved to 0.15 mm, 5.0 g soils were oxidized by potassium dichromate to measure soil organic matter (SOM) content by calculating the absorbance recorded at 590 nm with a spectrophotometry instrument (Jasco UV-visible, Tokyo, Japan). The aggregate distribution was determined using an auto sieve shaker (Orto Alresa Oass 203, Madrid, Spain) with a wet sieving method by Shi et al. [17]. Briefly, 3 sieves were applied to obtain aggregate size fractions: large macro-aggregates (>2000 μm), small macro-aggregates (250–2000 μm), micro-aggregates (53–250 μm) and silt + clay factions (<53 μm). The program was set to 500 rpm for 20 min at 10 s intervals. After the water flowing from the bottom became colorless, soils at different particle diameters were collected and dried at 80 °C until they reached a constant weight. Thereafter, soils of different particle diameters were weighed accurately.

### 2.4. Soil DNA Extraction, PCR and Processing of the High-Throughput Sequencing Data

DNA was extracted from 0.50 g of soil using the FastDNA SPIN Kit for soil (MP Biomedicals, Irvine, CA, USA) according to the manufacturer’s instructions. The DNA extracts were dissolved in 50 mL of TE buffer, and were qualified by gel electrophoresis. After that, the extracted DNA was evaluated by a micro-spectrophotometer (NanoDropND-2000, Thermo Fisher Scientific, Waltham, MA, USA) for quantity and quality and stored at −20 °C until further usage.

PCR amplification was conducted with primer set 519F (3′-CAGCMGCCGCGGTAATWC-5′) and 907R (3′-CCGTCAATTCMTTTRAGTTT-5′) targeting the V4-V5 region of the bacterial 16S rRNA gene. The 5-bp bar-code oligonucleotides were fused to the forward primer. Each PCR 50 uL reaction mixture contained 1.25 μM of deoxynucleosidetriphosphate, 2 μL (15 μM) of forward and reverse primers, 2 μM of Taq DNA polymerase (TaKaRa, Kusatsu, Japan), and 1 μL (50 ng) of genomic community DNA as a template. Each reaction was performed in triplicate to counter biases generated during amplification. The negative control was always run with sterile water as the template instead of soil DNA extract. Amplifications were carried out using the thermal conditions as follows: 94 °C for 5 min, 30 cycles (94 °C for 30 s, 55 °C for 30 s, and 72 °C for 45 s), and a final extension at 72 °C for 10 min. Reaction products for each sample were pooled and purified using the QIA quick PCR Purification Kit (Qiagen, Germany), and quantified using a NanoDrop ND-2000 micro-spectrophotometer. 

The sequences were quality-filtered and clustered into operational taxonomic units (OTUs) at 97% shared identity with consensus taxonomy. The taxonomic classification was based on the Silva 119 database (http://www.arb-silva.de/, accessed on 16 March 2022). 

### 2.5. Statistical Analyses

Differences in soil properties between afforested and non-afforested plots were compared using *T*-test (*p* < 0.05) using mean values by SPSS 19.0 (IBM Corporation, Somers, NY, USA). Figures of soil physicochemical properties were conducted with Origin 2021 (Origin Lab, Northampton, MA, USA). The diversity and richness indices were estimated with normalized OTU data using Mothur software. Microbial community data and the edaphic variable data (pH, BD, EC, AK, TN, AP, and SOM) were Hellinger standardized and log (x + 1)-transformed, respectively, for further multivariate analysis. A cluster analysis was used to analyze the soil microbial community structure based on Bray-Curtis similarities. A detrended correspondence analysis was conducted to identify the community data distribution model. Due to the gradient length value of <3.0 (linear model), we used a redundancy analysis (RDA), with a Monte Carlo permutation test (number of permutations: 999) for the comparison of the community structure and the correlation of the explanatory variables (R: vegan and ggplot2). Network analysis was conducted with the Molecular Ecological Network Analysis Pipeline (MENAP, http://ieg4.rccc.ou.edu/mena, accessed on 24 March 2022) [18,19]. A similarity matrix of the Pearson correlation coefficient was generated based on the OTU abundance data. Random matrix theory was then applied to automatically identify a similarity threshold for network construction. The networks were visualized with Cytoscape software. The module hubs, network hubs, and connectors were defined according to Deng et al. [18].

## 3. Results

### 3.1. Changes of Soil Attributes following T. Zhongshanshan Afforestation

**Figure 2:**
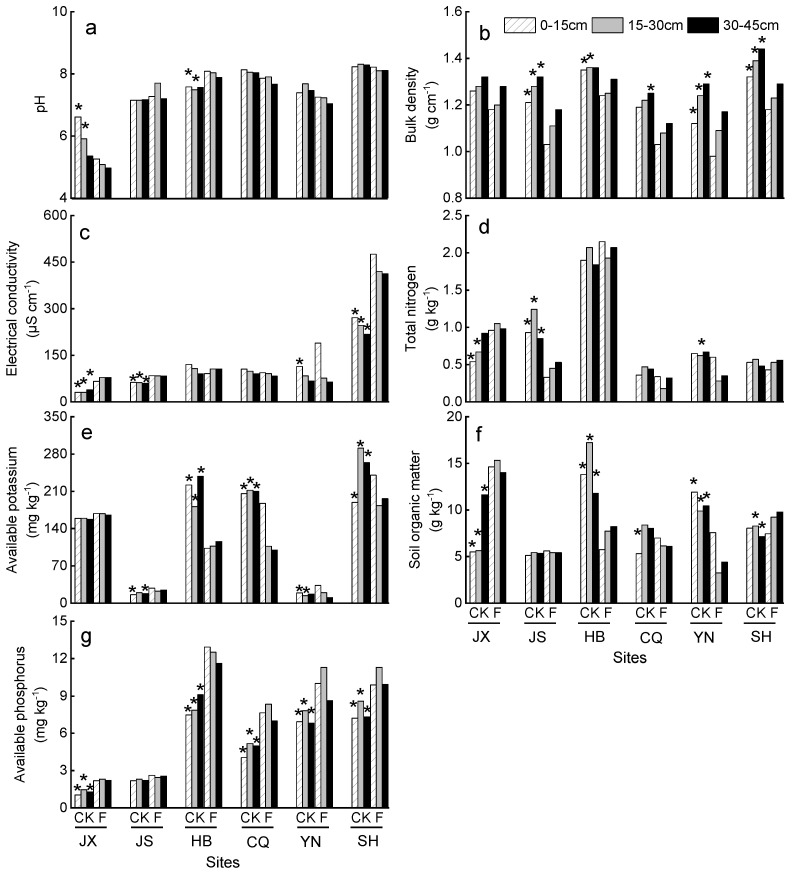
Soil properties in different *T*. Zhongshanshan plantations with increased age. Lowercase letters in the upper left corner represent pH (**a**), bulk density (**b**), electrical conductivity (**c**), total nitrogen (**d**), available potassium (**e**), soil organic matter (**f**), available phosphorus (**g**), respectively. The asterisks on the column indicate significant differences (*p* < 0.05) between CK and F of the same layer in each site. JX = Jiangxi; JS = Jiangsu; HB = Hubei; CQ = Chongqing; YN = Yunnan; SH = Shanghai; CK = non-forested soils; F = forested soils.

Concerning the plantations’ span, soil pH showed large divergences in the YRB. Soils in JX are acidic, and the others remain neutral or slightly alkaline (Figure 2). According to the soil nutrient grading criterion of the second national soil census of China [20], TN and SOM of HB were the highest among all sites, which was divided into grade I-II (>1.50 g kg^−1^) and IV (10–20 g kg^−1^), indicating a nutrient-rich soil condition. Conversely, CQ soils exhibited a lower TN (grade VI, <0.5 g kg^−1^) than all the other sites. AP of JX and JS was divided into grade VI (<3.00 mg kg^−1^), which denoted that P contents and supplies were poor. AK of SH (grade I, > 200 mg kg^−1^) was higher than all the other sites, and the rest ranged as CQ > HB > JX > JS > YN. 

For soil pH, significant differences (*p* < 0.05) between CK and forested soils (F) were observed in 0–15 cm and 15–30 cm of JX as well as HB, and 30–45 cm of JS (Figure 2a). BD of all soil depths differed significantly between CK and F in JS, YN and SH. In HB, BD of 0–15 and 15–30 cm also decreased significantly because of *T*. Zhongshanshan plantation (Figure 2b). Increase in soil EC was most evident in all depths in JX, JS, and SH (Figure 2c). TN in 0–15 and 15–30 cm of *T*. Zhongshanshan plantation in JX was 1.8 and 1.5 times higher than that in CK. JS and YN soils followed the opposite pattern as JX, exhibiting a reduction of TN due to *T*. Zhongshanshan growth (Figure 2d). In HB and CQ, a marked decrease was found in soil AK after *T*. Zhongshanshan growth in all soil depths. In YN, soil AK in the 0–15 and 15–30 cm increased by 1.7 and 1.3 times than that in CK, but decreased 37.7% in 30–45 cm. AK in SH soils followed opposite trends as compared to YN, since soil AK in the top 0–15 cm significantly increased but decreased by 30.4% and 25.4% in 15–30 and 30–45 cm than that in the corresponding depths in CK (Figure 2e).

Significant (*p* < 0.05) differences of SOM between CK and F were observed in JX, HB, YN and SH. Among the four above sites, *T*. Zhongshanshan plantation significantly (*p* < 0.05) increased SOM in JX and SH. However, in all depths in HB and YN, soils of *T*. Zhongshanshan plantation had lower (*p* < 0.05) SOM than non-forested soils (Figure 2f). In all plantation sites (except for JS), *T*. Zhongshanshan resulted in an enrichment of soil AP (Figure 2g). Generally, with regards to the effects of *T*. Zhongshanshan plantation, wide ranges of soil property divergences were observed in comparison with CK. 

**Figure 3:**
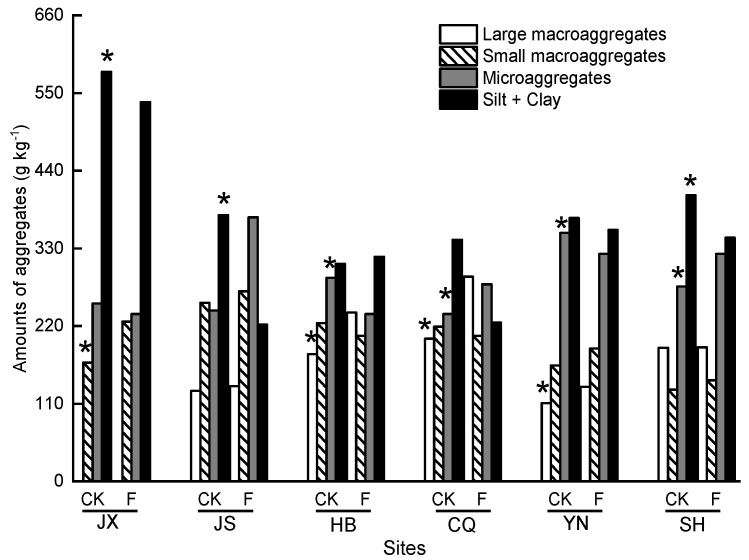
Amounts of aggregates in different *T*. Zhongshanshan plantations with increased age. The asterisks on the column indicate significant differences (*p* < 0.05) between CK and F in each site. JX = Jiangxi; JS = Jiangsu; HB = Hubei; CQ = Chongqing; YN = Yunnan; SH = Shanghai; CK = non-forested soils; F = forested soils. The asterisks on the column indicate significant differences (*p* < 0.05) between CK and F in each site.

Soils were composed most of silt + clay, with the highest levels recorded in JX in both CK (580 g kg^−1^) and F (557 g kg^−1^). In all plantation sites (except for HB and YN), the establishment of *T*. Zhongshanshan plantation resulted in a reduction (*p* < 0.05) of silt + clay compared with CK, with the highest 40.4% in SH, and then 34.2% in CQ. Conversely, soils under *T*. Zhongshanshan plantation had higher (*p* < 0.05) large macro-aggregates in HB, CQ and YN compared with CK (Figure 3). 

### 3.2. Changes of Soil Bacterial Community Structure following T. Zhongshanshan Afforestation

**Figure 4:**
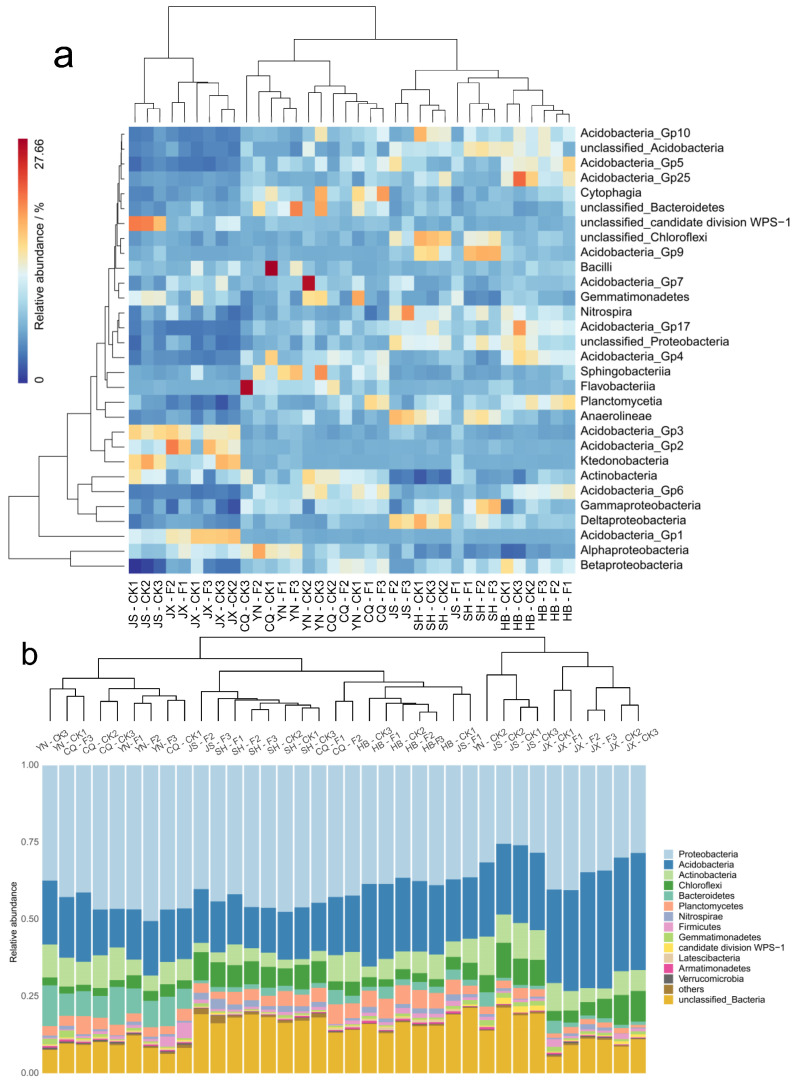
Cluster analysis and relative abundance of bacterial class (**a**) phylum (**b**) based on Bray-Curtis similarities in forested and non-forested soils. JX = Jiangxi; JS = Jiangsu; HB = Hubei; CQ = Chongqing; YN = Yunnan; SH = Shanghai; CK = non-forested soils; F = forested soils. Arabic numbers of 1, 2 and 3 after abbreviations indicated soil depth at 0–15, 15–30 and 30–45 cm, respectively.

The heatmap analysis for the top 30 most abundant bacterial classes showed that soils of the same site clustered in one group, and *T*. Zhongshanshan plantation separated from that of non-forested sites (Figure 4a). This finding indicated that afforestation had a different bacterial community composition. Overall, the 3 most abundant classes in CK were *Flavobacteriia*, *Acidobacteria*_Gp25 and *Sphingobacteriia*, while afforestation of *T*. Zhongshanshan significantly (*p* < 0.05) increased the relative abundance of the classes *Flavobacteriia*, *Acidobacteria*_Gp5, and *Bacilli*. The dominant phyla (more than 25% of the gene sequences) were Proteobacteria, Acidobacteria, and Actinobacteria in all soils (Figure 4b). The hierarchical clustering results showed that the composition of the soil bacterial communities in *T*. Zhongshanshan plantation separated from that of non-forested sites. *T*. Zhongshanshan plantation significantly increased (*p* < 0.05) the relative abundance of the phyla Proteobacteria and Nitrospirae, on the contrary, decreased the abundance of Acidobacteria and Gemmatimonadetes (*p* < 0.05, Figure 4b).

**Figure 5:**
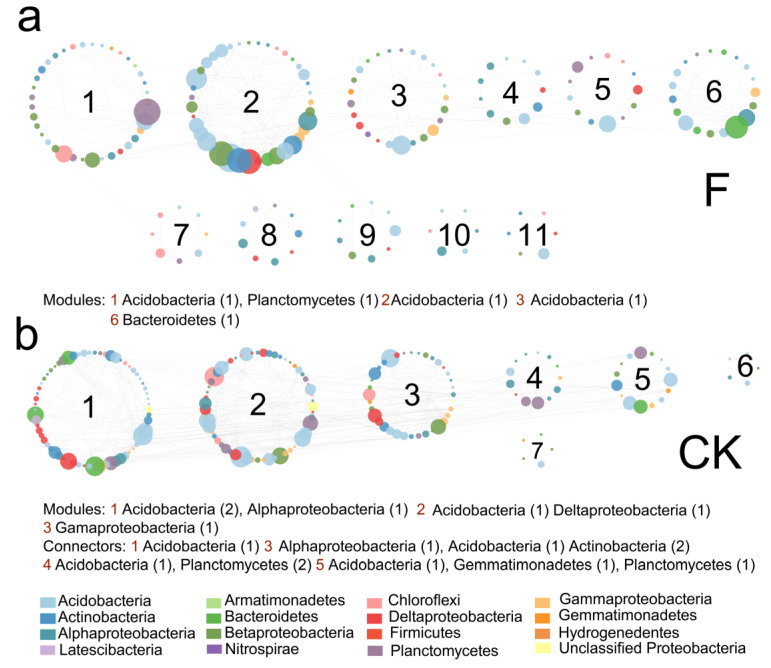
The structure of F (**a**) network and CK (**b**) network. CK = non-forested soils; F = forested soils. Modules with fewer than 5 members are not displayed. Circle colors represent the OTU node-affiliated taxa at the bacterial phylum level (Proteobacteria are at the class level) and the circle size represents the connectivity degree of the nodes. Grey lines represent positive correlations and red lines represent negative correlations. The numbers in the parentheses are the number of module hubs or connectors.

The soil microbial network of the *T*. Zhongshanshan plantation sites had more modules (31 vs. 19) and modularity (0.765 versus 0.516) than the non-forested soils, which reflected the more microbial structure complexity of the former. The non-forested sites had the connectors while they disappeared after the *T*. Zhongshanshan plantation, maybe resulting in the increase of average path distance in the *T*. Zhongshanshan plantation. In addition, the module hubs changed from groups of Acidobacteria, *Alphaproteobacteria*, *Deltaproteobacteria* and *Gamaproteobacteria* in non-forested sites to Acidobacteria, Planctomycetes and Bacteroidetes in *T*. Zhongshanshan plantation sites. In the *T*. Zhongshanshan plantation network, module 1, module 2, and module 3 had the same module hub Acidobacteria (Figure 5a). In the non-forested network, modules 1 and 2 had the same hub Acidobacteria (Figure 5b).

The Shannon diversity indices decreased with increasing soil depth among all plantation sites. Except for SH and YN, soils under *T*. Zhongshanshan plantation showed higher (*p* < 0.05) Shannon diversity than those of CK, especially in JX, JS, and HB, where each soil depth all exhibited significant (*p* < 0.05) differences. Compared with the middle reaches (HB and JX, Shannon: 9.02 and 8.84) and the lower reaches (JS and SH, Shannon: 8.82 and 9.06), *T*. Zhongshanshan plantations in the upper reaches (YN and CQ, Shannon: 9.10 and 9.10) of the Yangtze River had a relatively higher microbial biodiversity. The Simpson diversity indices among all sites exhibited no significant differences. In all sites but SH, planting *T*. Zhongshanshan trees significantly (*p* < 0.05) increased the Chao1 richness (Table 2). 

### 3.3. Correlations between the Soil Properties and the Microbial Community Composition

**Figure 6:**
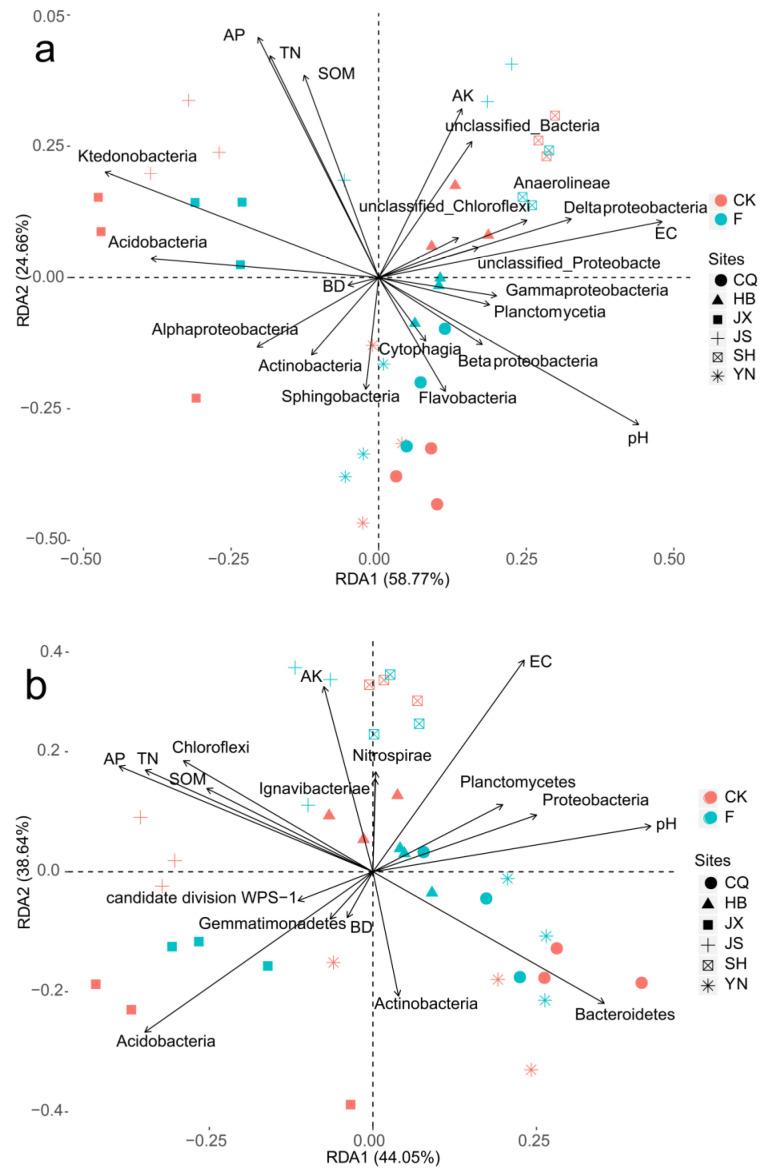
Redundancy analysis (RDA) based on the bacterial class (**a**) and phylum (**b**) data. JX = Jiangxi; JS = Jiangsu; HB = Hubei; CQ = Chongqing; YN = Yunnan; SH = Shanghai; CK = non-forested soils; F = forested soils; BD = bulk density; EC = electrical conductivity; AK = available potassium; TN = total nitrogen; AP = available phosphorus; SOM = soil organic matter.

The redundancy analysis (RDA) model based on soil bacterial class and phylum genera level satisfactorily distinguished the *T*. Zhongshanshan plantation sites from each other and from the CK (Figure 6a,b). RDA also showed that JS, and JX group together along the main RDA1 axis according to their microbial community. Permutation tests for the RDA model manifested that the RDA was enough to explain the correlation between the microbial community structure and the soil edaphic variables (*p* < 0.01). In class level, AK, pH and EC were positively correlated with RDA1 axis (*r* = 0.46, *p* < 0.01; *r* = 0.46, *p* < 0.001; *r* = 0.99, *p* < 0.001), while AP, TN and SOM exhibited negative correlation (*r* = −0.52, *p* < 0.01; *r* = −0.51, *p* < 0.001; *r* = −0.42, *p* < 0.001) with RDA1 explained the 58.77% of variance (Figure 6a). Soil pH, AK and EC were positively correlated with class *Anaerolineae*, *Deltaproteobacteria*, *Gammaproteobacteria*, *Planctomycetia*, *Betaproteobacteria*, *Flavobacteriia,* and *Cytophagia* (Figure 6a). 

In phylum level, pH and EC were positively correlated with RDA1 axis (*r* = 0.95, *p* < 0.001; *r* = 0.54, *p* < 0.001), while AK, AP, TN, and SOM exhibited negative correlation (*r* = −0.10, *p* < 0.01; *r* = −0.92, *p* < 0.001; *r* = −0.91, *p* < 0.001; *r* = −0.84, *p* < 0.001) with 44.0% of variance explained (Figure 6b). Except for BD, soil properties could significantly influence the bacterial community structure both phylum and genera level (*p* < 0.05). At the phylum level, soil EC, and pH were positively correlated with Actinobacteria, Bacteroidetes, Planctomycetes, and Proteobacteria (Figure 6b).

## 4. Discussion

### 4.1. Effect of T. Zhongshanshan Plantation Establishment on Soil Attributes

Compared to the soils in non-forested sites, our study proved that *T*. Zhongshanshan plantation ameliorated most of soil attributes, e.g., TN in JX and SH and AP in HB, CQ and YN. This meant that *T*. Zhongshanshan plantations had relatively replenished the nutrient pools in soils of the YRB. With increasing plantation age, the development of understory vegetation formed an important component of net primary productivity, and their life cycle provided an important input of carbon (C), N, and other nutrients. The significant increase in AP content was directly related with the SOM. Reports on changes in the chemical properties of soils caused by the conversion of former agricultural lands into forests were diverse, both positive and negative. Forest plantations in southern Rwanda proved that soil C and N accumulated over time in the *Eucalyptus saligna* afforested sites [21]. Likewise, Mishra et al. [22] observed an increase in total soil C and N during 9 years of *E*. *tereticornis* plantation. In a chronosequence study of *Pinus* plantation in Australia, Turner et al. [23] observed a 35% loss in SOM after 10 years of establishment. Cross-country studies of *Pinus* showed either a decline or increase in soil SOM in tropical regions [24]. However, the soils in JS and YN under *T*. Zhongshanshan plantations had relatively lower TN compared to CK. According to our previous pot experiment, *T*. Zhongshanshan plants exhibited soil-specific characteristics, preferring soils rich in N, P, and K [17]. The initial decline of soil TN in *T*. Zhongshanshan plantation might result from a restrained supply of K, since plants demand more nutrients to satisfy photosynthesis, mainly through adenosine triphosphate (ATP) synthesis. In the 1–10 year range of plantation ages, fast-growing *T*. Zhongshanshan plants in JS and YN could compete for soil resources with other plants and soil organisms. This phenomenon would be especially noticeable in the YRB if the soil elements, like P and K, were in relatively inadequate supply. SOM in HB and YN belonging to *T*. Zhongshanshan plantations also showed a tendency of reduction. Same tree species or species within a functional group can differ in their effects on soils. In the cases where decreases in soil C of plantations were significant, Hailu [25] attributed the differences to impacts of plantation density. In this study, *T*. Zhongshanshan in HB and YN was initially planted at 1111 stem·ha^−1^ (3 m spacing between and within rows) and had not yet been thinned. As forest ages, a larger initial tree density may gradually decrease the light transmittance for understory plants and lead to fierce competition, which in turn is not conducive to C availability to soils. Silvicultural interventions in *T*. Zhongshanshan plantation can create a more spacious canopy, and then soils may be more coupled with light penetration to increase temperatures, which is instrumental in litter decomposition.

In this study, BD of the *T*. Zhongshanshan plantation was significantly lower than of the non-forested sites (except for JX). This may be attributed to a dense root system that is capable of efficiently penetrating the soil substrate, thus increasing soil porosity and reducing BD. However, *T*. Zhongshanshan plants in JX were sparse and relatively small, whose root penetration was not as extensive as that of the > 6-year-old plantations, resulting in no significant differences of BD as compared to adjacent non-forested soils. The influence of forest cover on increasing soil porosity, by reducing BD and increasing capillary and gravitational pores is well evidenced [26]. The shift from relatively small aggregate sizes to large, afforestation of *T*. Zhongshanshan had a positive influence on physical characteristics principally at the expense of soil macro-aggregates augment. Concordant with our finding, a conspicuous increase in large macro-aggregates of the *Robinia pseudoacacia* afforestation in the Loess Plateau was also recorded [27], highlighting the crucial role of afforestation in improving soil stability. Wei et al. [28] reported a marked decline in macro-aggregate amounts within 50 years of converting forest to cropland, compared with a notable increase after long-time afforestation. Soil structures may be re-established to pre-deforestation standards by consecutive vegetation restoration. Afforestation over time increases root biomass, thus favoring the formation of macro-aggregates. 

### 4.2. Effect of T. Zhongshanshan Plantation Establishment on Soil Bacterial Community

Afforestation can affect soil nutrients by changing the nature of litter and root exudates, both of which can alter the growth of soil microbes and ultimately transform soil bacterial diversity [29]. Afforestation of *T*. Zhongshanshan significantly increased the relative abundance of the class *Flavobacteriia*, *Acidobacteria*_Gp5, and *Bacilli*. This implied that the community composition of *Flavobacteriia*, *Acidobacteria*_Gp25 and *Sphingobacteriia* changed during the transformation from non-forested soils to forested soils, which was owing to changes of soil physicochemical properties in *T*. Zhongshanshan plantations. The influence of plants on the microbiome class had been previously reported. Long-term rice cultivation orderly changed the succession of the bacterial class community by increasing relative abundances of *Anaerolineae*, *Deltaproteobacteria*, *Betaproteobacteria*, and *Gammaproteobacteria,* which were capable of decomposing carbohydrates [30]. Furthermore, Proteobacteria and Acidobacteria were the most 2 abundant phyla in YRB soils, akin to the findings in other soils and environments [31,32]. Our results suggested that the establishment and development of *T*. Zhongshanshan plantation will lead to alteration in the understory microhabitat, acting as the driving force to increase the relative abundance of the phyla Proteobacteria and Nitrospirae, on the contrary decrease the abundance of Acidobacteria and Gemmatimonadetes. Proteobacteria belong to copiotrophic groups and are considered to be r-strategists that preferentially utilize easily accessible sources of C or alternatively closely associated with the soil C cycle. Acidobacteria belong to oligotrophic groups and prefer nutrient-poor environments. The arresting shift in soil bacterial community compositions following *T*. Zhongshanshan afforestation suggested that these below ground communities transitioned from slow-growing oligotrophic groups to fast-growing copiotrophic groups. Similar to what we perceived, Liu et al. [33] concluded that the *R*. *pseudoacacia* plantation improved the soil nutrient conditions according to higher Proteobacteria abundance.

Shannon index and Chao1 richness of soil bacteria were higher for afforested sites, this can be attributed to changes in the relative abundance of Proteobacteria, which were reported to represent the most abundant bacterial phylum. This increase in diversity could result from higher above-ground biomass in *T*. Zhongshanshan plantation soil as compared to CK, and understory microhabitats further mediate the diversity and composition of the soil microbial community. Diverge from Zhao et al. [34], whose study manifested that soil bacterial diversity increased sharply in the initial stages of *R*. *pseudoacacia* plantations, our results did not exhibit such a clear tendency. The research of *R*. *pseudoacacia* plantations was carried out in the Wuliwan Watershed, where is semi-arid and the soil is deposited by wind erosion. This discrepancy suggested that not only afforestation species, but also variable factors may influence soil bacterial diversity, including soil types and climate. 

**Figure 7:**
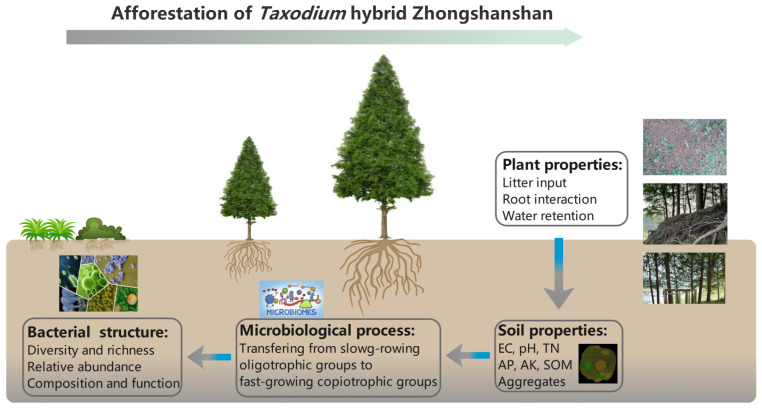
Afforestation of *Taxodium* hybrid Zhongshanshan influences soil bacterial community structure by altering soil properties.

Afforestation played a momentous role in the soil bacterial community and potential functional composition due to substantial contributions to the soil system. In our study, the soil properties explained 85.21% and 82.69% of the respective variation in the soil bacterial phylum and class level and could significantly influence the bacterial community structure. The relationship of afforestation on the soil microbial community has been well documented in a variety of field-based studies [35,36]. In Horqin Desert afforested with *Pinus sylvestri*s, Cao et al. [36] demonstrated that soil properties were regarded as indicators of the bacterial community and function. SOM is notably the primary factor influencing the soil microbial community at the phylum level. Liu et al. [37] reported that *R*. *pseudoacacia* afforestation shaped the soil bacterial community composition via enhancement of soil physicochemical properties, of which soil AP was the predominant factor. Moreover, soil bacterial diversity in non-forested lands was proved highly susceptible when compared with that in afforested lands, suggesting that afforestation is expected to increase biodiversity stability by enriching the soil nutrient pool, mainly N, SOM and P [38]. These findings confirmed our inference that the soil bacterial composition and diversity were affected by soil properties following *T*. Zhongshanshan afforestation (Figure 7).

## 5. Conclusions

Afforestation of *T*. Zhongshanshan plantation had greatly improved soil bacterial abundance and diversity, which had been accompanied by increases in soil nutrients, and a decrease in soil bulk density. It forecasted that *T*. Zhongshanshan plants established a new forest ecological balance through changing the soil environment and indicated that the metabolic pattern changed from resource acquisition to resource utilization. With the extension of forest areas in the YRB, we suggested that measures such as thinning or pruning should be implemented by forestry sectors with increasing plantation age, which could maximize soil C and nutrient contents. In nutrient-depleted soils, proper fertilization is recommended for this fast-growing species to achieve a high biomass production and replenishment of the nutrient pools. Potential risks of *T*. Zhongshanshan plantation for reduced soil fertility over time, such as leaching of nutrients from the soil, immobilization of nutrients in the organic layer and biomass should be further studied.

## Figures and Tables

**Table 1:** General information of *T*. Zhongshanshan plantations.

Sites	Age	Soil Colour	Coordinate	Mean Height (m)	Mean DBH (cm)	CD (m × m)	Density (Stems ha^−1^)	AGB (t ha^−1^)
JX	6	red	116°7′4″ E 28°30′49″ N	7.7	10.7	1.6 × 2.2	1111	20.3
JS	8	yellow-brown	118°49′35″ E 32°10′59″ N	8.9	12.6	1.9 × 2.6	1111	34.8
HB	9	yellow-brown	112°13′37″ E 30°19′34″ N	10.4	14.1	2.9 × 4.0	1111	61.9
CQ	11	purple	108°27′3″ E 30°45′58″ N	13.4	17.1	4.4 × 6.1	920	131.7
YN	13	yellow-red	102°46′41″ E 24°49′43″ N	13.7	23.3	5.6 × 6.1	1111	180.1
SH	14	yellow	121°38′29″ E 31°34′57″ N	14.2	22.7	6.4 × 7.2	830	152.6

JX = Jiangxi; JS = Jiangsu; HB = Hubei; CQ = Chongqing; YN = Yunnan; SH = Shanghai; DBH = diameter at breast height; CD = Crown diameter; AGB = aboveground biomass.

**Table 2:** Shannon, Simpson and Chao1 indexes of soil bacteria in different *T*. Zhongshanshan plantations with increased age.

Sites	Treatments	Depth (cm)	Shannon	Simpson	Chao1
JX	CK	0–15	8.22b	0.99a	1513b
		15–30	8.14b	0.99a	1646b
		30–45	8.06b	0.99a	1639a
	F	0–15	8.92a	0.99a	1723a
		15–30	8.98a	0.99a	1966a
		30–45	8.64a	0.99a	1570a
JS	CK	0–15	8.34b	0.99a	1957a
		15–30	8.19b	0.99a	1721b
		30–45	7.80b	0.99a	1329b
	F	0–15	8.90a	1.00a	1869a
		15–30	8.87a	1.00a	2063a
		30–45	8.71a	0.99a	1957a
HB	CK	0–15	8.67b	0.99a	2261b
		15–30	8.60b	0.99a	2036b
		30–45	8.40b	0.99a	1879a
	F	0–15	9.04a	1.00a	2534a
		15–30	9.08a	1.00a	2524a
		30–45	8.96a	1.00a	2316a
CQ	CK	0–15	8.92a	0.99a	2296a
		15–30	8.64b	0.99a	2328a
		30–45	8.42b	0.99a	2009b
	F	0–15	9.13a	1.00a	2298a
		15–30	9.10a	1.00a	2342a
		30–45	9.07a	1.00a	2382a
YN	CK	0–15	9.01a	1.00a	1922b
		15–30	9.01a	1.00a	2157b
		30–45	8.98a	1.00a	2194b
	F	0–15	9.20a	0.99a	2395a
		15–30	9.15a	0.99a	2565a
		30–45	8.96a	0.97a	2476a
SH	CK	0–15	9.03a	1.00a	2210a
		15–30	9.06a	1.00a	2205a
		30–45	9.05a	1.00a	2194a
	F	0–15	9.11a	1.00a	2262a
		15–30	9.09a	0.99a	2327a
		30–45	8.99a	0.99a	2102a

JX = Jiangxi; JS = Jiangsu; HB = Hubei; CQ = Chongqing; YN = Yunnan; SH = Shanghai; CK = non-forested soils; F = forested soils. Different lowercase letters in each column indicate significant differences (*p* < 0.05) between CK and F in the same layer.
